# Developing a Low-Cost IoT-Based Remote Cardiovascular Patient Monitoring System in Cameroon

**DOI:** 10.3390/healthcare11020199

**Published:** 2023-01-09

**Authors:** Tagne Poupi Theodore Armand, Md Ariful Islam Mozumder, Sikandar Ali, Austin Oguejiofor Amaechi, Hee-Cheol Kim

**Affiliations:** 1 Institute of Digital Anti-Aging Healthcare, Inje University, Gimhae 50834, Republic of Korea; 2 Department of Information and Communication Technology, The ICT University, Cameroon Campus, Yaoundé P.O. Box 526, Cameroon; 3 College of AI Convergence, Institute of Digital Anti-Aging Healthcare, u-AHRC, Inje University, Gimhae 50834, Republic of Korea

**Keywords:** remote patient monitoring, cardiovascular disease, IoT-based application, lean UX process

## Abstract

(1) Background: Cameroonians are exposed to poor health services, more so citizens with cardiovascular-related diseases. The global high cost of acquiring healthcare-related technologies has prompted the government and individuals to promote the need for local research and the development of the health system. (2) Objectives: The main goal of this study is to design and develop a low-cost cardiovascular patient monitoring system (RPM) with wireless capabilities that could be used in any region of Cameroon, accessible, and very inexpensive, that are able to capture important factors, well reflecting the patient’s condition and provide alerting mechanisms. (3) Method: Using the lean UX process from the Gothelf and Seiden framework, the implemented IoT-based application measures the patients’ systolic, diastolic, and heart rates using various sensors, that are automated to record directly to the application database for analysis. The validity of the heuristic evaluation was examined in an ethnographic study of paramedics using a prototype of the system in their work environment. (4) Results: We obtained a system that was pre-tested on demo patients and later deployed and tested on seven real human test subjects. The users’ task performances partially verified the heuristic evaluation results. (5) Conclusions: The data acquired by the sensors have a high level of accuracy and effectively help specialists to properly monitor their patients at a low cost. The proposed system maintains a user-friendliness as no expertise is required for its effective utilization.

## 1. Introduction

The World Health Organization (WHO) estimated that death from cardiovascular diseases (CVDs), in 2019, represent 32% of all global deaths. Of these deaths, 85% were due to heart attack and stroke [1]. Heart and blood vessel illnesses, collectively, are referred to as CVDs. Working on behalf of the World Heart Federation (WHF), the Pan-African Society of Cardiology [2], in a 2020 report, concluded that 11.85% of total deaths in Cameroon are caused by the cardiovascular disease-related condition. At a time when the protracted anglophone crisis has forced the closure of numerous healthcare facilities, the COVID-19 outbreak has exposed Cameroon’s fragile healthcare system [3,4]. The most famous cardiac centre of Cameroon, the Cardiac Centre of Shisong (CCS), located in the north-west region and currently the lone cardio-surgical centre, has been facing severe operation difficulties in the middle of the socio-political crisis affecting the region, thus worsening the condition of many cardiac patients all over the country [5]. Additionally, this is not unique to Cameroon, Nef et al. reported, in a recent paper, that during the COVID-19-related lockdown in Germany, a substantial rise in cardiovascular mortality was found [6]. So far, there is only a little research on heart disease informatics from Cameroon. The world is moving toward a system where illnesses are detected in real-time. The RPM or telemonitoring is categorized as remote healthcare. The most important part of remote healthcare is the use of technology to monitor patients outside of hospital settings. However, as Craig et al. [7] stated, the efficacy of the complex interventions, such as telemonitoring, depends on the context in which they are applied. With over 80 percent of Cameroon people residing in rural areas; any such technology will be highly valued. There are a few hospitals with modern equipment, but they are expensive for individuals living in remote areas. Moreover, doctors are reluctant to travel to those remote places and provide care for the locals. However, practically every village has access to the Internet. This issue served as inspiration for the system that was proposed, which will help the patients because they may utilize the real-time monitoring system to prevent heart attacks, take their medications on schedule, and consult doctors from home.

According to Lakmini, Naeem and Keshav’s [8] study, IoT-based health monitoring systems have a number of benefits, including ongoing patient monitoring, real-time illness detection, a reduction in the need for and cost of hospitalization, technology use, emergency medical aid, and lastly, more accurate data collection.

Published guidance [9] from the American Heart Association (AHA) supports the use of remote patient monitoring for patients with cardiovascular diseases. The AHA determined that the technologies used for remote patient monitoring contribute to better cardiovascular disease outcomes and concluded that the RPM is a cost-effective and value-enhancing solution. Brahmbhatt and Cowie [10] classified the RPM, according to the following categories:

- Structured telephone support for patients: this is generally provided by specialists of the heart failure (HF) team; it assists in a disease management program or a post-discharge service;
- Standalone RPM systems: These devices can be used at home by patients to measure some physical parameters. e.g., BP, heart rate, weight, and oxygen saturation;
- Remote monitoring using cardiac implantable devices: These are used to collect real-time data to aid the HF management. They include devices, such as pacemakers and ICDs, implanted primarily for therapeutic purposes;
- Wearable devices: They include patches, watches, or textiles equipped with sensors that can monitor a patient’s parameters e.g., ECG, BP, body temperature, blood glucose concentration, and body posture.

**Figure 1:**
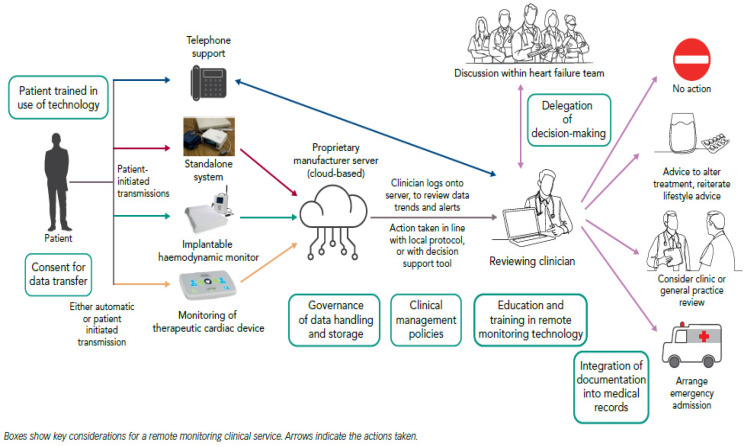
Remote monitoring system.

In the Brahmbhatt and Cowie [10] description of a typical remote monitoring system (see Figure 1), the trends in collected data or movement of variables outside the pre-set limits may be used by the control team, which could be a HF team for heart-related diseases to trigger a variety of actions, including a telephone call or recommendations on lifestyle and medication changes, or a clinic review for further assessment, or even urgent admission to hospital.

This paper presents the research and development of a low-cost IoT-based remote cardiovascular disease patient monitoring system adapted to Cameroon’s technological level and IT infrastructure, as well as those of other Sub-Saharan African (SSA) countries with similar IT development levels, that accessible and very inexpensive that are able to capture important constants, well reflecting the patient’s condition and provide alerting mechanisms. The validity of the heuristic evaluation was examined in an ethnographic study of paramedics using a prototype of the system in their work environment. The remainder of this paper is structured as follows: In Section 2 we present related research while Section 3 describes the materials and methods, Section 4 describes the results and discussions, and Section 5 provides the conclusion of the presented work.

## 2. Related Work

Dohr et al. [11] proposed a blood pressure monitoring system. The device collecting data was known as keep in touch (KIT) and was only for healthcare services. The system was also made for a mobile application. Following the transmission, the data could be viewed online, and monitoring was possible, thus all of the variations of the BP were notified. Suh et al. [12] proposed a remote patient monitoring system for CHF. The design and implementation were carried out following a three-tier architecture, consisting of pervasive biosensors, a web server, and a back-end database helping guardians with feedback via text messages or emails. Prajoona et al. [13] proposed an IoT-based health monitoring system. The heartbeat sensor is based on the principle of photoplethysmography. It measures the change in the volume of blood through any organ of the body which causes a change in the light intensity through that organ. The digital pulses are given to a microcontroller for calculating the heartbeat rate. The IoT server puts away the computerized information that is transmitted through the Zigbee protocol to the neighborhood server Zigbee, to store the data. If the patient does not have any past therapeutic record, then the server makes a new ID and stores the information in its database.

Chao et al. [14], following the IoT architecture, proposed a remote monitoring system for heart disease. A sensing device was built and connected to the connecter (android smartphone) in which a Java application was designed to receive raw data (heart rate, BP, ECG, and SpO2) and transmit it to the server-side, following four transmission modes: (1) Real-time continuous transmission for all data. (2) Continuous transmission in special periods. (3) Event-triggered transmission, (4) Transmission on patients’ demand. Mamidi et al. [15] proposed a RPM system able of detecting heart attacks with the help of monitoring the heart rate and blood pressure, based on the IoT. Every user will be wearing a device that can be placed on one wrist that contains a sensor that helps to monitor the heart rate and even the blood pressure, and can detect and raise an alarm of a heart attack occurrence. Senthamilarasi et al. [16] designed a smart patient health monitoring system using the IoT. In the system, a mobile device-based wireless healthcare monitoring system was developed, which can provide real-time online information about the physiological conditions of a patient. The patient’s temperature, heartbeat rate, and EEG data are collected. The read values are then transferred to the PC via a RS232 Bluetooth module by URL and the data is received in the database as transmitted to the doctor’s smartphone, containing the application for analysis.

A heart attack detection monitor for heartbeat sensing using the Internet of Things, by Gurjar and Neha [17], consists of a sensor interfaced to an ATMEGA 328 microcontroller that allows for checking the heart rate and temperature reading and then transmitting them over the Internet. In this system, the user could set both the upper and lower limit values for the collected heartbeat. Any value out of range will cause an alert. Brahmbhatt and Cowie [10], in their recent studies, confirmed that the IoT-enabled remote monitoring and screening of heart failure (HF) patients’ health conditions anywhere and anytime, is rapidly evolving and it is one of the most advanced approaches in telecardiology, regarding feasibility and clinical evidence. Sethuraman [18] proposed an IoT-based system for heart rate monitoring and heart attack detection. The real-time monitoring is carried out via Adafruit, this platform is more secure to store the information and uses the MQTT protocol, which has lots of advantages over others. Gopi and Punarselvam [19] proposed a heart attack recognition and heart rate monitoring system using the IoT layered architecture. A plug-and-play heart rate sensor is used here to collect the patient data and send it to the back-end application. As the other systems, an alert is sent to the physician as a notification of the patient’s parameters.

In Cameroon, Arthur Zang developed a commercialized version of a remote monitoring device called the cardiopad, to assist cardiologists and medical personnel in carrying out medical exams from remote locations [20]. The cardiopad was able to connect with some sensors to collect the patient data and transmit it to another cardiopad for expert analysis through a GSM network, as the device was equipped with a SIM card unit.

The system proposed by Roman et al. [21] consists of an early wearable prototype for health monitoring, that measures the body temperature, heart rate, and heart rate fall detection, to show these data on a liquid crystal display (LCD) screen and send notifications to the caregiver using a GSM module. Our proposed system includes more functionalities, such as the server-based applications and appropriate feedback from cardiologists, while focusing on blood pressure parameters: a key indicator of heart diseases.

## 3. Materials and Methods

### 3.1. Study Design

We adopted the user-centred design (UCD) process as a guide for constructing the proposed system. A heuristic evaluation was examined in an ethnographic study of paramedics using a prototype of the system in their work environment. The study design was designed to consist of 8–10 participants. The sample size of a maximum of ten participants was deliberately chosen, and this is considered sufficient, and it is an acceptable size, as proposed by Hwang and Savendy [22]. The participants were those available and who also met our predetermined criteria of importance. Cameroon is classed within what is generally accepted as a low-income and middle-income country (LMIC). It has a weak healthcare system and the unofficial number of available cardiologists and healthcare professionals, per citizen, is very low. Therefore, forming the required clinical expert panel in Cameroon was difficult and this study was fortunate to have experts who were particularly interested in the study’s low-cost design and who also see their profession as a social service responsibility. They had the motivation and focus to evaluate the product without remuneration and were considered experts because they satisfied the two basic Goldman’s properties [23]: (a) An expert knows a lot about a given topic; (b) An expert can apply that extensive knowledge of that given topic to other situations, so as to rationally predict their possible outcomes.

### 3.2. Specify the Usage Context

The user-centred design (UCD) technique [24], an iterative procedure with four stages, is used in this study: To specify the usage context, specify the requirements, produce the design solutions, and the evaluation. To situate the process sufficiently within the available resources, the lean UX process view of the UCD from Gothelf and Seiden [25], guided the research and construction. We created and used a semi-structured interview to find out the users’ use context with ten experts at Bamenda Regional Hospital, Bingo Hospital, and Shisong Hospital in the north-west region of Cameroon. These hospitals were selected because of the availability of qualified cardiologists and their willingness to assist in the system evaluation. In order to determine the qualities that the system should have, in order to support the healthcare monitoring of cardiovascular patients, we thematically evaluated the recordings. Among the most important discoveries were:

- Remote monitoring: Have a digitized history of patients’ vital signs that are monitored remotely in real-time over the internet;
- Alert notifications: When the system recognizes an uncommon circumstance, to generate and establish notifications for patients, cardiologists or caretakers, and family members;
- Medical record: The system must be capable of keeping the medical records and providing easy, secure access to them in real time;
- Communication with family: The system must offer a feature to provide in-depth summaries of the doctor’s recommendations to the patient or family, in order to encourage the family participation in patient care;
- The subsequent UCD phase was initiated, in light of those findings.

### 3.3. Specify Requirements

#### 3.3.1. System Architecture of the Cardiovascular RPM System

**Figure 2:**
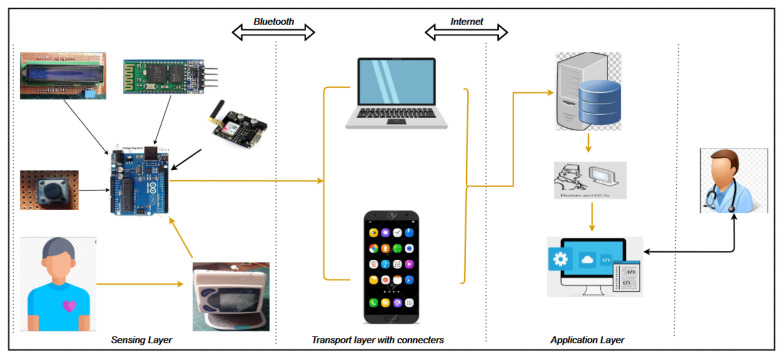
System architecture of the cardiovascular RPM system.

Figure 2 shows the whole system architecture of the cardiovascular RPM system. This system architecture consists of three parts: the sensing layer, the transport layer, and the application layer.

The sensing layer consists of different devices connected to the Arduino microcontroller. The devices communicate together to obtain the data needed for transmission to the transport layer, which is made up of a connecter (smartphone or laptop) through short-distance wireless communication (Bluetooth). For the pilot study, a simple phone set and laptop internet connectivity was used to facilitate the activities at the application layer. The application is linked to the database that holds the health-related data. Doctors can see and analyse the data of the patient, and this is considered as reports from the patients. Upon analysing the report of the patient, doctors give the important feedback and recommendations by prescribing prescriptions. The patient can speak to the doctor on a video call without coming to the hospital, if there is any urgency. In Cameroon, fast internet connectivity and constant electricity are a luxury. The system is demonstrated using a GSM module supporting a 2G network (reliable) that can transmit reliable data through SMS, as a backup option.

#### 3.3.2. Hardware Tools

The components needed to build the system were moderately assembled. The components that were used to construct the monitoring system are described in Table 1.

#### 3.3.3. Software Requirements

Pressman et al. [26] said that to solve an issue or accomplish a goal, a software program must have certain criteria or capabilities that must be included in the application, requested by a client, or gleaned from the context of the user. A functional requirement is a description of the service that the software must offer. The requirements that are directly linked to the RPM system and that describe its complete functionality include:

- Users must be authenticated in the RPM application;
- The latest information gathered by the vital signs sensors should be displayed to caregivers through the RPM application;
- A communication module must be included in the RPM application for the cardiologist and care team to use, when speaking with the patient or their family;
- The RPM application should allow the cardiologist and care team to access the clinical information of the patient as required.

The non-functional requirements are those that specify conditions or characteristics that the software must possess. The requirements, as well as the information from the interviews and project context analysis include:

- The device with the sensors for monitoring the vital signs should have Bluetooth Smart (BLE), to send the generated data to the database;
- The availability of the system must be near permanent and offer a decent service level for the users;
- The stored data can be consulted.

#### 3.3.4. Produce Design Solutions

We used the specifications and the architecture to develop a medium-fidelity RPM application prototype using mock-up tools. This project’s development was carried out in three phases:

- The connection of the Arduino Uno to all the other components;
- The implementation of the whole project on a solderless experiment board (breadboard);
- Obtaining the data and forwarding them to a connecter automatically, as a result of a Bluetooth/SMS request, or manually by pressing the pushbutton provided in the system.

**Figure 3:**
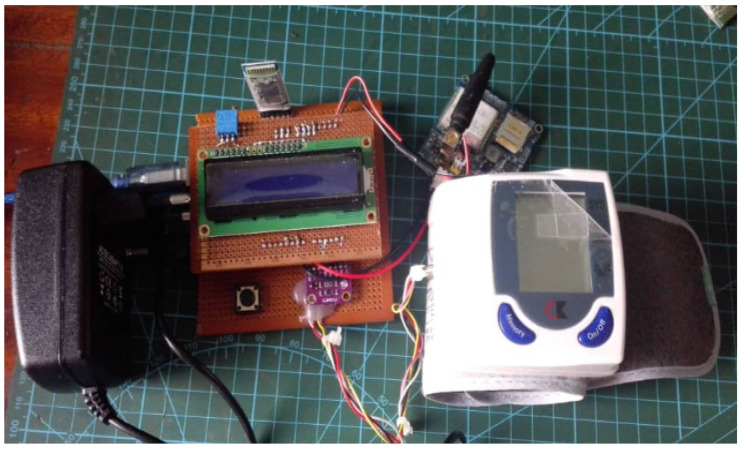
Prototype of various components connected with the RPM microcontroller.

Figure 3 shows the actual physical setup of the prototype.

**Figure 4:**
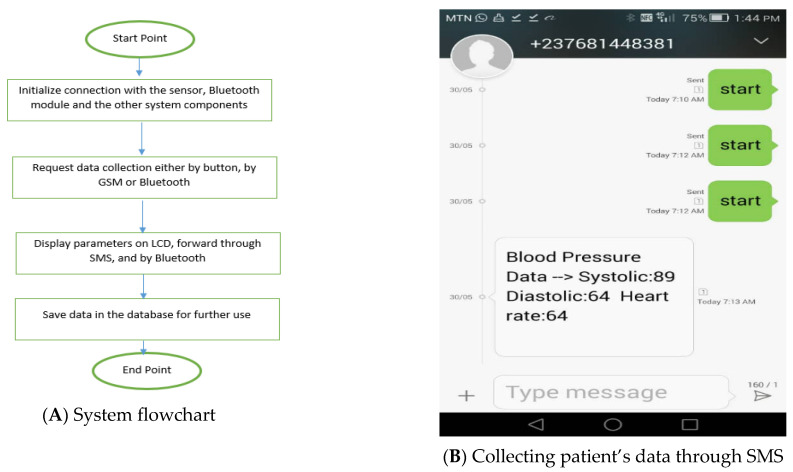

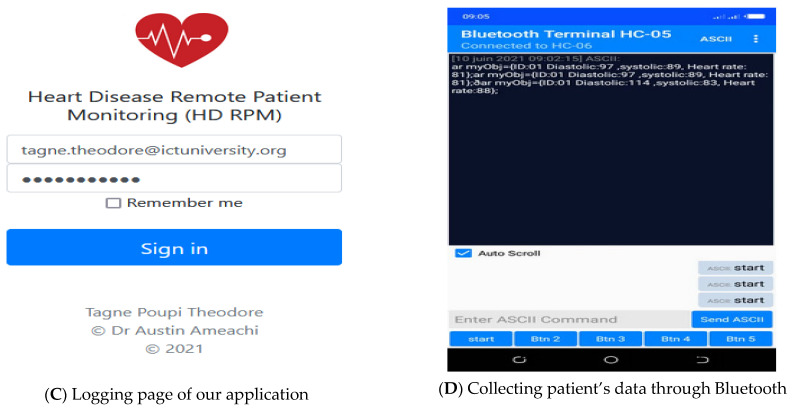
(**A**–**D**) shows respectively the prototype system flowchart, the screen for collecting the patient data and notifications, the logging page of our application, and collecting the patient data through Bluetooth interface.

Figure 4A–D shows respectively the prototype system flowchart, the screen for collecting the patient data and notifications, the logging page of our application, and collecting the patient data through Bluetooth interface. Our GSM module was instructed in such a way that when the message “start” is sent to the number in the GSM module, the controller starts the BP monitor and collects the parameters (systolic, diastolic, and heart rate). The collected parameters are now sent as a reply message to a number stored in the microcontroller a few seconds later. The number is supposed to be that of the doctor or/and the guardian. There is no reply if the device is not powered.

**Figure 5:**
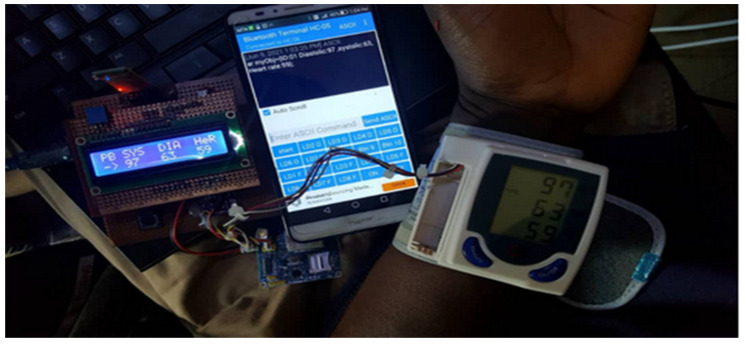
Confirmation of the correct data transmission.

Our application could interact with the device through a Bluetooth connectivity. To request for the patient parameters, the application sends a request message “start” to the Bluetooth module, the message starts the BP monitor, and the data is collected. The collected data is sent under the JSON (JavaScript object notation) format and saves it automatically in the database. An extra parameter is added to those sent through SMS; this is the device ID that will help to identify a patient in a multi patients’ system, since there is no human identification sensor available. The figure below shows how the data is requested through Bluetooth. Figure 5 shows the actual prototype setup of the confirmation of the correct data transmission or the communication screen.

**Figure 6:**
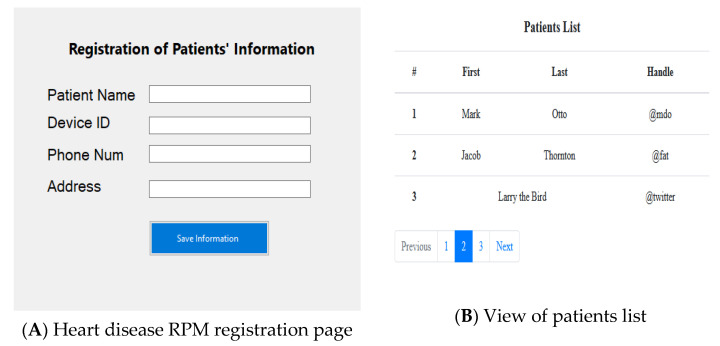
(**A**,**B**) shows the Heart disease RPM registration page and View of patients list, respectively.

With regards to the application interface, to create a communication with the sensor and server, a web application was developed to serve as an interface. Our application here is called “heart disease RPM” and permits the medical expert, herein a cardiologist, to access the patient’s data. The app’s welcome and registration pages are shown in Figure 6A. In order to create an account, the patient must fill out the form. Basic details including the patient’s full name, email address, phone number, residence, and age are required. When registering as a patient, certain conditions must be satisfied. The form carries a device ID or serial number that can only be assigned to a single patient, this helps in identifying to whom the data belong. The application user can view the list of available patients (see Figure 6B) and clicking on a patient’s name will display his report, showing all of the variations in the data collected. The physician can now message a patient to give him guidelines on how to proceed, from the recorded values.

**Figure 7:**
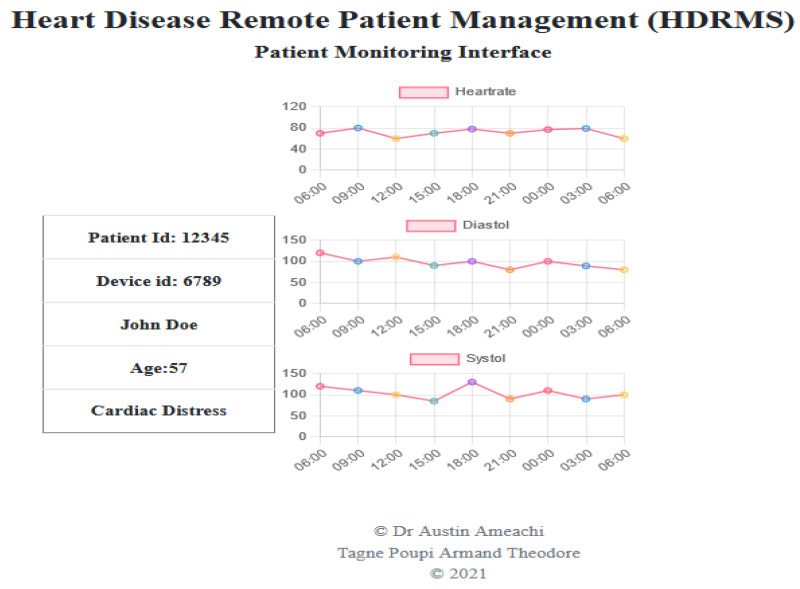
Patient’s records view from the DB.

A doctor can write the patient a prescription after seeing the patient’s report, as it is stored in the database (see Figure 7). The patient’s symptoms must be noted by the doctor. The doctor must then draft the prescription itself. 

## 4. Results and Discussion

Our design process led to the development of a low-fidelity prototype for the proposed solution. The initial low-fidelity prototype allowed the authors to merge and cement the design ideas. It further helped to generate additional plans for the design of the system and the component devices. Following the UCD process as a guide, we first identified our target audience. With that, we designed and conducted interviews with selected stakeholders to learn more about the populations affected by the need for accessible and cost-effective methods to gather vital signs. This process of designing and conducting interviews led to our development of unique personas that could potentially benefit from the solution. Our ideation process allowed us to review the strengths and weaknesses of the existing solutions. The results strengthen the benefits our proposed solution will bring to Cameroon’s practical aspects of healthcare.

To improve the acceptance of the developed system, we conducted a heuristic assessment study using an expert review of the RPM prototype, to make sure that the needs discovered through the contextual study were incorporated in a way that would be simple for caregivers and patients to use. In our evaluation, we focused on the parameters collected from the sensor. The evaluation of the output lies at the level of data transmission, the BP monitor is equipped with a display unit, if the transmission is accurate then the values on the BP monitor are going to match with those from the LCD of our microcontroller, and the Bluetooth signal sent to the mobile phone. Upon initialization, when the values are received by the microcontroller, there is a display on the LCD unit to confirm the values read by the sensor. The values demonstrate the data integrity in the transmission.

To make sure everything was understood, the evaluators had a brief discussion on the problems’ severity scale and heuristics, prior to each evaluation. Ten evaluators in total participated in the evaluation procedure at three different locations. To prevent biases and to guarantee independent outcomes, each system evaluator conducted the evaluation independently. The following heuristics principles were considered for evaluation: (1) Visibility of the system status; (2) Match between the system and the real world; (3) User control and freedom; (4) Consistency and standards; (5) Error prevention; (6) Recognition rather than recall; (7) Flexibility and efficiency of use; (8) Aesthetic and minimalist design; (9) Help users recognize, diagnose, and recover from errors; (10) Help and documentation. According to Nielsen [27], the values for the ratings were between 0 (not a problem) and 5 (catastrophe). Each expert had an evaluation form with the heuristics and descriptions of the potential issues to be discovered, as well as a severity rating for each issue or item.

Table 2 shows the experts’ classification of the problem’s severity. A critical problem was identified by an expert and no evaluator identified problems as catastrophe.

Most of the usability problems identified were related to labelling, user interface navigation, aesthetic and minimalist design, variations in responsiveness, and response time. The UI’s navigation was frequently criticized by participants. The resolution of the usability problems resulted in a significant increase in the perceived usability, efficiency, and effectiveness of the system before its official release to interested hospitals in Cameroon.

*Strength*

The proposed system helps patient to save money or is cost-effective and allows for the greater accessibility for patients living in rural areas or those with limited mobility. It makes healthcare more available. The RPM allows doctors to reach out to potential patients. While many people cannot stand going to clinics or, for certain reasons, cannot afford to visit a doctor, the RPM will bring fast and reliable consultations with a physician right to their homes.

Our proposed system showed many advantages over the existing one. With the cardiopad, the collected data were transmitted through a GSM network to the cardiologist for analysis, disabling the data transmission out of the country. Our system is IoT-based, and the server is accessible, not just in Cameroon, but wherever there is internet connectivity, making the data analysis possible for cardiologists all over the world.

According to a report from the WIPO (World Intellectual Property Organization) [28], the cardiopad was designed and commercialized for about 2200 euros, which is significantly cheaper than other commercially available, but not affordable by all health centers or individuals in the country. We proposed a cheaper system, affordable to our people and accessible to individuals. Our system makes use of a mobile smartphone, which is common for Cameroonians to own. The system also maintained a user-friendliness and does not require any medical or IT expertise while used.

*Limitations*

An essential component in a RPM system is an adequate security protocol; however, our basic software components are not yet sufficiently developed to accommodate a secure and efficient wireless network.

Due to the availability of the resources and the complexity of the task, we were not able to provide the ECG sensors in our system. Extending this study by including ECG data sensors with data visualization, would be important.

## 5. Conclusions

The design and execution of a remote health monitoring system for patients with cardiovascular-related conditions, are presented in this research. Cardiovascular diseases (CVDs) are a group of disorders of the heart and blood vessels. According to the WHO, the constant observation of the patient can greatly reduce their discomfort. Figure 1 shows a schema that illustrates some key issues that require discussion before a fully functioning remote monitoring service is comprehensively established. In Cameroon, the availability of remote monitoring applications will be an innovation. In the Cameroon market, there is a significant demand and supply gap, which presents an opportunity for solutions, such as ours, to act and alter the situation for the benefit of our population. Despite the cheap cost of our application, most hospitals in Cameroon will require sufficient financial and structural support from the government, nongovernmental organizations (NGOs), and other institutions, in order to finance such a minor investment. With this low-cost application, a new age of consumer-driven health has arrived in Cameroon, with great future benefits in cardiovascular disease prevention, diagnosis, and management. The following areas of this research can be improved in the future: (1) Include a cross-platform functionality, such as iOS support; (2) Include more sensors, such as blood glucose, digital scales, and activity sensors; (3) video calling and chatting features; (4) ECG data sensor with data visualization; (5) to overcome security issues, incorporate blockchain technology; (6) integration with electronic health records; and (7) establishing logistics for patient onboarding and staff education.

## Figures and Tables

**Table 1:** Description of the components.

Components	Description
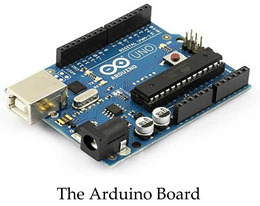	Arduino is a microcontroller board, that contains a board power supply, a USB port to communicate with the PC, and an Atmel microcontroller chip. We decided to use Arduino Uno, based on the board size, pricing comparison, and connectivity, making our IoT-based RPM system suitable for it.
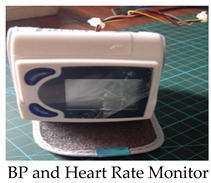	This is the main monitoring device of our system. It is responsible for collecting the data (diastolic, systolic, and heart rate) using its sensors to send to the Arduino UNO through wires. Following our interview with the medical experts, it was adopted that the normal BP values depend on two factors: age and sex. The normal range for BP and HR measurements was given, based on the factors.
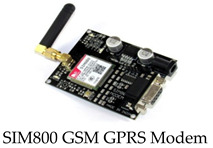	This device has many features, but the ones we may look at are: connection to any global GSM network with any 2G SIM; the possibility to make and receive voice calls using an external 8Ω speaker and electret microphone; capability of sending and receiving SMS messages. This module was optional for our system, our application will be able to perform its function, that is sending SMS messages, but because of the quality of the internet connectivity, we thought it wise to have this module and a 2G SIM card to send and read the parameters. By doing this we are sure that even if the connectivity fails with the connecter, the medical expert and guardian can receive a message on the real-time physical condition of the patient.
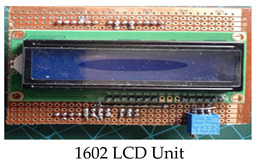	This is a liquid crystal display unit attached to a microcontroller. The role of the LCD unit in our system is to display the values from the sensing device, to confirm the correctness of the transmitted data.
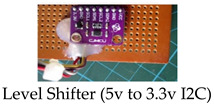	This was needed to reduce the voltage received from the microcontroller.
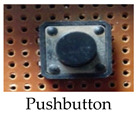	Our pushbutton picks the parameters on the patient’s demand without any wireless connectivity (Bluetooth and GSM) instruction.
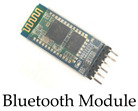	We decided to use a HC-05 Bluetooth module to link our sensing layer device to the smartphone or computer. All data collected by the sensor are transmitted through Bluetooth under the JSON (JavaScript object notation) format.
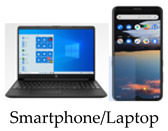	These devices are used in the second layer of our IoT design (the intermediate layer) as connectors.

**Table 2:** Number of problems and their classification, according to the experts.

Classification	Bingo–Location 1	Shisong–Location 2	Bamenda–Location 3	Total
Can be improved	5	7	8	**20**
Minor problem	0	1	1	**2**
Serious problem	4	0	1	**5**
Critical problem	1	0	0	**1**
Catastrophe	0	0	0	**0**
**Total**	**10**	**8**	**10**	**28**
